# Expression Analysis of the Mediators of Epithelial to Mesenchymal Transition and Early Risk Assessment of Therapeutic Failure in Laryngeal Carcinoma

**DOI:** 10.1155/2019/5649846

**Published:** 2019-12-06

**Authors:** Nora Kariche, Nabila Moulaï, Leila-Sarah Sellam, Samir Benyahia, Wahiba Ouahioune, Djamel Djennaoui, Chafia Touil-Boukoffa, Mehdi Bourouba

**Affiliations:** ^1^Department of Cell and Molecular Biology, Team Cytokines and Nitric Oxide Synthases. Faculty of Biology, University Houari Boumediene USTHB, Algiers, Algeria; ^2^Central Laboratory for Anatomopathology, Frantz Fanon Hospital, Blida, Algeria; ^3^Oto-Rhyno-laryngology Department, Mustapha Pacha Hospital, Algiers, Algeria

## Abstract

Laryngeal squamous cell carcinoma (LSCC) is an aggressive malignancy which lacks early predictors of prognosis. Here, we hypothesized that expression and prognostic characterization of the critical mediators of epithelial to mesenchymal transition (EMT) may provide key information in this regard. Linear regression and multiple correspondence analyses were performed on immunohistochemical data obtained from 20 invasive tumors. Principal component and unsupervised hierarchical clustering were used to analyze the dataset patterns associating with LSCC metastatic profile. Survival and death risk assessments were performed using Kaplan–Meier and hazard ratio tests. Data mining analysis using CHAID decision tree and logistic regression analysis was applied to define the predictive value of the risk factors of tumor aggressiveness. Our analyses showed, that in invasive LSCC tumors, cells associating with a mesenchymal profile were likely to exhibit enhanced NOS2, TGF-*β*, and IL-17A expression levels, concomitantly to NF-*κ*B nuclear translocation. IHC data deciphering determined that EMT induction was also linked to the enrichment of the tumors with CD68+ populations and IL-10 signal. Strikingly, dataset cluster analysis showed that these signatures could define distinct patterns of invasive tumors, where NOS2 associated with IL-10 expression, and TGF-*β* and IL-17A signals associated with MMP-9 activation. Decision tree analysis identified IL-17A as a possible predictor of LSCC aggressiveness. Altogether, our results show that distinct immunological patterns would support the acquisition of EMT features in invasive LSCC and suggest that IL-17A may be useful in the early identification of patients “at-risk” of therapeutic failure.

## 1. Introduction

Laryngeal squamous cell carcinoma (LSCC) is a highly metastatic malignancy of the head and neck caused by tobacco and alcohol intake [1]. The disease is characterized by a dismal prognosis and to the best of our knowledge, absence of predictors of therapeutic failure which may help in improving disease management [2]. This is mainly due to the complexity of the molecular aspects of resistance to therapy and the difficulties in identifying predictive molecular markers of tumor aggressiveness and lethality by traditional statistical approaches.

Tumor invasion occurs in the context of smoldering inflammation as a consequence of phenotypical alterations which affect tumor epithelial cells adhesion and attachment to the extracellular matrix (ECM) [3]. This process occurs consecutively to a partial to a full transition of tumor cells from an epithelial to a mesenchymal phenotype, in a process known as epithelial-mesenchymal transition (EMT) [4]. The downregulation of epithelial cadherin (E-cadherin) represents a critical step in epithelial tissue architecture disruption [5]. Loss of E-cadherin has been shown to promote the release of membranous *β*-catenin to mediate, dedifferentiation and activation of the expression of the mesenchymal marker, vimentin [5, 6]. These modifications are believed to constitute to date a key event regulating the metastatic process [6]. Other than structural changes, the loss of E-cadherin/*β*-catenin complex may contribute to cancer progression by modifying a complex network of pathways that tightly regulate fundamental processes as oxidative stress, immune evasion, and cell metabolism [7–9].

Despite the fact that EMT in head and neck cancers has recently been associated with metastasis [10], little attention has been given to date to the possible prognostic value of its mediators [11–13]. Interestingly, in recent years, a number of soluble mediators of EMT among which, the reactive nitrogen/oxygen intermediates (RNI/ROI), matrix metalloprotease 9, the immunomodulatory cytokine IL-10, and TGF-*β*, as well as the proinflammatory cytokines IL-6 and IL-17A, have gained increased attention due to their possible value in cancer management [13–17]. Yet, to the best of our knowledge, the importance of these biomarkers in LSCC has remained to date unexplored. Here, we thought to examine through an integrative analysis and their possible association to the acquisition of a mesenchymal profile and determined their possible influence on LSCC lethality risk.

## 2. Materials and Methods

### 2.1. Study Subjects and Biological Material Preparation

Twenty LSCC patients (16 males and 4 females, median age 62 years) and 9 age-matched healthy donors (HD, 7 males, and 2 females) were enrolled at the M. Pacha Hospital between 2012 and 2013. All patients were at stage III-IV based on the TNM staging (UICC 2002). The tumor was located in the glottis in 13 patients. The tumor invaded the glottis and the supraglottic area in 5 patients and affected the three laryngeal regions in 2 cases. Tumor samples were collected prior to chemotherapy or radiotherapy.

The study was approved by the ethics committee of the National Agency for Research Development in Health (ATRSS). Written informed consent was obtained from all subjects before participating in the study. Specimen collection was performed prior to treatment. Biopsies were fixed in 10% formol, dehydrated, and embedded in paraffin. HE-stained sections were evaluated for the presence of invasive LSCC. Adjacent normal epithelium and in-situ carcinoma analysis were used for comparisons. Peripheral blood was collected on lithium heparin and centrifuged at 2,000 rpm for 10 min. Plasma was collected and stored at −20°C until use.

### 2.2. Immunohistochemistry (IHC)

Tissue sections of 5 *μ*m were tested against E-cadherin (NCH-38, prediluted format, Dako), *β*-catenin (17C2, prediluted format, Novocastra), vimentin (V9, 1/500 Novocastra), NOS2 (SAB4502012, 1/1000, Sigma), NF-*κ*B (p65, 2A12A7, 1/500, Invitrogen), TGF-*β* (TB21, 1/1000, Thermofisher), IL-6 (10C12, 1/100, Novocastra), IL-17 (50104, 1/100, Invitrogen), IL-10 (945A2A5, 1/100, Invitrogen), CD68 (KP1, prediluted format, Dako), and MMP-9 (2C3, 1/200, Santa Cruz Biotechnology). Formalin-fixed, paraffin-embedded sections were deparaffinized, hydrated, and treated using a high temperature for antigen retrieval in EnVision™ FLEX Solution (Dako). After blocking of endogenous peroxidase, sections were incubated 1h at 37°C with appropriate primary antibodies. Immunorevelation and counterstaining were done with EnVision™ FLEX (Dako). The sections were observed and photographed using an Olympus microscope CX41 equipped with a DP21 Olympus digital camera.

### 2.3. IHC Scoring

All scoring methods were based on reported literature. E-cadherin, *β*-catenin, and NOS2 scores were assessed following Acs et al. [18] by an evaluation of the estimate of the percentage of stained cells (%SC) and the intensity of staining scored on a 4-tiered scale intensity of staining (IS; 0–3) (IHC score = IS × %SC). Vimentin scores were expressed as the percentage of labeled tumor epithelial cells. The signal in the stromal and the endothelial cells was taken as an internal control [19]. For epithelial IL-6 and nuclear NF-*κ*B, stainings were scored 0 or 1 [20]. TGF-*β* expression was scored for intraepithelial IS [21]. Since IL-6, IL-17A, IL-10, CD68, and MMP-9 stainings were located in tumor and stromal areas, the markers were quantified in both compartments; Stromal IL-6 signal was scored as % of stained cells at high-power field (HPF). IL-17A, CD68, and MMP-9 signals were evaluated as % of stained cells in different epithelial and stromal HPFs [22]. Stromal IL-10 positivity was quantitatively expressed as % of stained cells in HPF. IHC score was used for tumoral IL-10 [23]. For all slides, 5 representative microscopic fields were randomly and independently evaluated by two investigators.

### 2.4. Griess Reaction

Nitric oxide (NO) in plasma was assessed by nitrites (NO_2_^−^) quantification using Griess reaction [24].

### 2.5. Enzyme-Linked Immunosorbent Assay

Plasmatic cytokines levels were determined at 450 nm using ELISA kits (Invitrogen). Sensitivity levels were 2 pg/mL for IL-6 and IL-17A, <1 pg/ml for IL-10, and 15.6 pg/ml for TGF-*β*.

### 2.6. Zymography

Plasmatic metalloproteinase activity was assessed in all our samples by gelatin zymography after protein concentration normalization. Following denaturation under nonreducing conditions and electrophoresis (10% gel, 0.2% gelatin), the samples were renatured in 2.5% Tx-100 and incubated in 50 mM Tris-HCl (pH 7.4), 5 mM CaCl2, and 20 mM NaCl buffer for 17 h at 37°C. The gels were stained with Coomassie blue (R250) and destained in 10% acetic acid/40% methanol. Gelatinolytic bands intensity were evaluated using ImageJ.

### 2.7. Statistical Analysis

All data were tested for normality using the D'Agostino–Pearson Omnibus test. One-way ANOVA test was used to compare the means of more than two groups followed by the Bonferroni's multiple comparisons test. Mann–Whitney or unpaired-t-test were used to compare the means two groups and Spearman or Pearson correlation tests were carried out for bivariate correlations. Continuous data were expressed as mean ± standard deviation and *p* values ≤0.05 were accepted as statistically significant. Data were analyzed with GraphPad Prism 6.0.1.

### 2.8. Survival Analysis and Data Mining

All compared patients received a similar combined regimen of cisplatin, fluorouracil, and docetaxel. Overall survival (OS) over 36 months was estimated using Kaplan–Meier and log-rank test. The relative risks (RR) were assessed using the Cox proportional hazards regression model. Forest plots were done using GraphPad Prism. Chi-squared automatic interaction detection (CHAID) was used for decision tree prediction of resistance to therapy. Data were analyzed with XLstat 2017.7.

### 2.9. Multivariate Analysis

Multiple correspondence analysis (MCA) was used to test the relation between EMT and the immunological biomarkers. The qualitative variables were ranked in function of the % positive cells as follows: NOS2, E-cadherin, *β*-catenin, and TGF-*β*, 0 ≤ 5%, 1 = 5–25%, 2 = 25–50%, and 3 = 50%; Vimentin 0 < 5%, 1 = 5–25%, and 2 > 25%; IL-17A 0 <1%, 1 >1–5%, and 2 > 5%; NF-*κ*B 0-cytoplasmic and 1-nuclear; IL-6 0-negative and 1-positive. Principal component analysis (PCA) was used to identify the principal components which accounted for the majority of the variation within the dataset. All quantitative variables were centered and normalized before test.

### 2.10. Hierarchical Clustering and Heat Mapping

Heat mapping and unsupervised hierarchical clustering were performed using centered and normalized data with Genesis 1.8.1.

## 3. Results

### 3.1. EMT Features Are Prominent in Invasive LSCC

**Figure 1:**
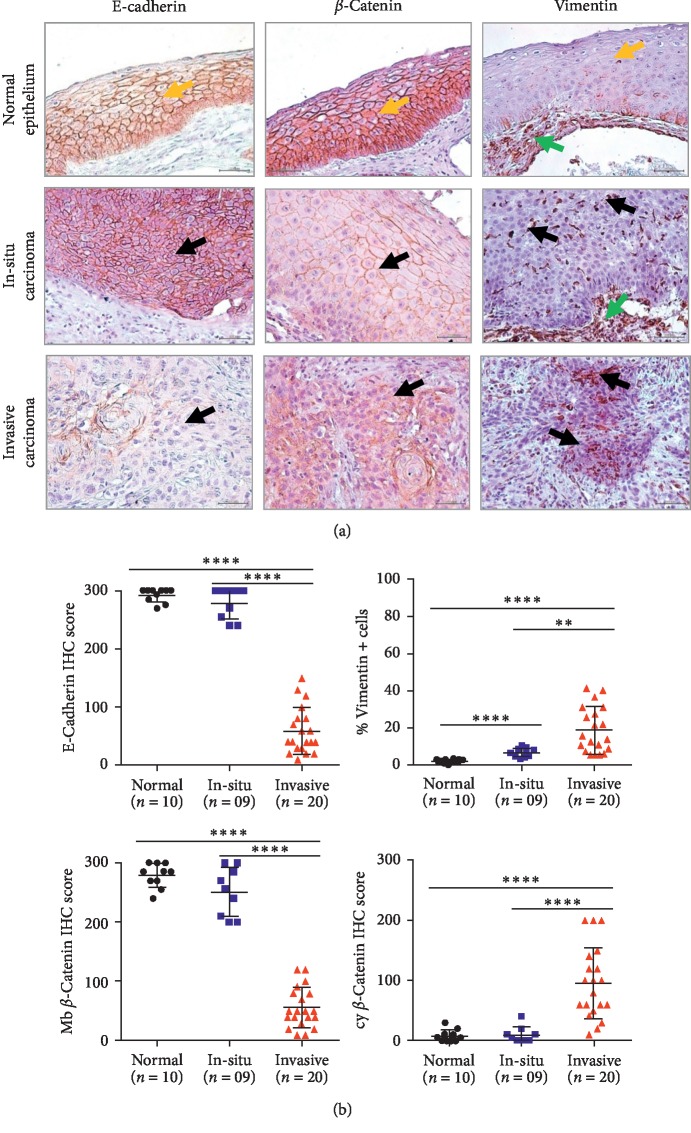
Epithelial-mesenchymal transition is prominent in invasive LSCC. (a) Representative immunohistochemical sections for EMT markers expression in normal epithelium, in situ carcinoma and invasive carcinoma (magnification ×40) (yellow arrow: normal epithelium, black arrow: tumor cells, and green arrow: stomal cells). (b) Immunohistochemical scoring comparison for EMT markers expression in normal epithelium (*n* = 10), in situ carcinoma (*n* = 9), and invasive carcinoma (*n* = 20). Data are shown as mean ± SD. One-way ANOVA test was used for multiple group comparisons (^*∗∗∗∗*^*p* ≤ 0.0001) followed by Bonferroni's multiple comparison test (^*∗∗*^*p* ≤ 0.01).

First, we examined the expression of EMT markers (E-cadherin, *β*-catenin, and vimentin) in invasive LSCC (*n* = 20), adjacent in-situ carcinoma (*n* = 9), and normal epithelium (*n* = 10). Representative immunohistochemical sections for EMT markers expression are shown in Figure 1(a). Interestingly, loss of E-cadherin and gain of cytoplasmic *β*-catenin and vimentin were observed in invasive carcinoma compared with adjacent in-situ carcinoma and normal epithelium (Figure 1(b)).

### 3.2. NOS2-Associated NF-*κ*B Activity Is Linked to EMT Induction in Invasive LSCC

**Figure 2:**
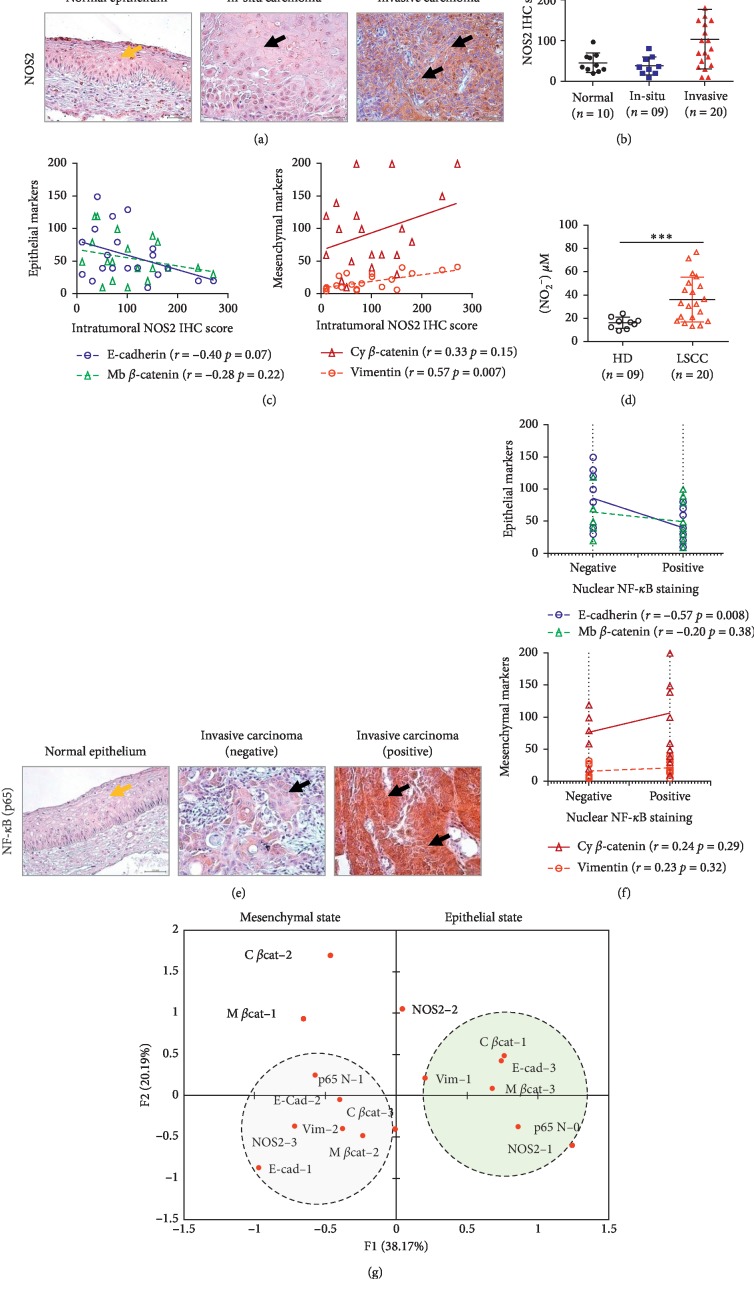
Epithelial-mesenchymal transition is prominent in invasive LSCC tissues under NOS2/NF-*κ*B inflammatory conditions. (a) Representative immunohistochemical sections for NOS2 expression in normal epithelium, in situ carcinoma, and invasive carcinoma (magnification ×40) (yellow arrow: normal epithelium and black arrow: tumor cells). (b) Immunohistochemical scoring comparison for NOS2 expression in normal epithelium (*n* = 10), in situ carcinoma (*n* = 9), and invasive carcinoma (*n* = 20). Data are shown as mean ± SD. One-way ANOVA test followed by Bonferroni's multiple comparison test. (c) Pearson correlation analysis between NOS2 and EMT markers expression in invasive LSCC (*n* = 20). (d) Plasmatic NO_2_^−^ levels in healthy donors (HD) (*n* = 9) and invasive LSCC patients (*n* = 20) were assessed using the Griess method, and *t*-test was performed for statistical analysis; data are shown as mean ± SD. (e) Representative immunohistochemical sections for NF-*κ*B (p65) expression in normal laryngeal tissues and in invasive carcinomas (magnification ×40) (yellow arrow: normal epithelium and black arrow: tumor cells). (f) Pearson correlation analysis between NF-*κ*B (p65) nuclear status and EMT markers expression in invasive LSCC (*n* = 20). (g) MCA of the relation between EMT, NOS-2, and NF-*κ*B (p65).

Production of nitric oxide (NO) via nitric oxide synthase (NOS2) plays a key role in inflammation-dependent head and neck cancer progression [25]. Yet, that production has shown to exert controversial effects on EMT [26, 27]. To explore if a possible link prevailed between NOS2 with EMT in LSCC, we analyzed the profile of association of NOS2 with EMT by IHC. To our surprise, we observed that the tumoral NOS2 signal was enhanced in the invasive tissues in comparison with the normal epithelium and in-situ carcinoma (*p* ≤ 0.0001, Figure 2(a)-2(b)). Interestingly, NOS2 upregulation strongly associated with a shift from an epithelial to a mesenchymal profile (NOS2/E-cad: *r* = −0.41, *p*=0.07; NOS2/Vim: *r* = 0.57, *p*=0.007; Pearson) (Figure 2(c)). Of note, a concomitant increase in NOS2 dependent nitrites synthesis was also observed in patients with invasive LSCC (Figure 2(d)). We concluded that NOS2 mediated chronic inflammation is positively associated with EMT in invasive LSCC.

Considering that NOS2 activity can influence EMT through signaling via NF-*κ*B [28], we next tested the occurrence of these events in the invasive tumors. As most of the invasive tissues displayed nuclear NF-*κ*B immunostaining (60%) (Figure 2(e)), we observed with interest that presence of a trend towards a moderate correlation between NOS2 to an active NF-*κ*B signal (*r* = 0.38, *p*=0.1; Pearson). In turn, nuclear NF-*κ*B signal significantly associated with loss of E-cadherin expression (*r* = −0.57, *p*=0.008) (Figure 2(f)). A multiple correspondence analysis (MCA) was next conducted to determine the patterns of association occurring between EMT biomarkers, NOS-2, and NF-*κ*B (p65 N) status. Data analysis revealed the presence of two factorial axes explaining 58.36% of the total inertia. A dual pattern particularly segregated along the first eigenvector and represented 38.17% of the total variance. On the positive side of the axis, tumors with a strong epithelial pattern clustered together and associated with variables indicating low levels of tissular inflammation and NF-*κ*B activation. By contrast, on the negative side of the axis, a cluster of variables indicating a pronounced mesenchymal profile cosegregated with variables relating to enhanced NOS2 signaling and NF-*κ*B activation (Figure 2(g)). Taken together, these data suggested that NOS2/NF-*κ*B activity would significantly be linked to EMT induction in invasive LSCC.

### 3.3. IL-17A/TGF-*β* Enriched LSCC's Inflammatory Microenvironment Prevail to EMT Induction in Invasive LSCC

**Figure 3:**
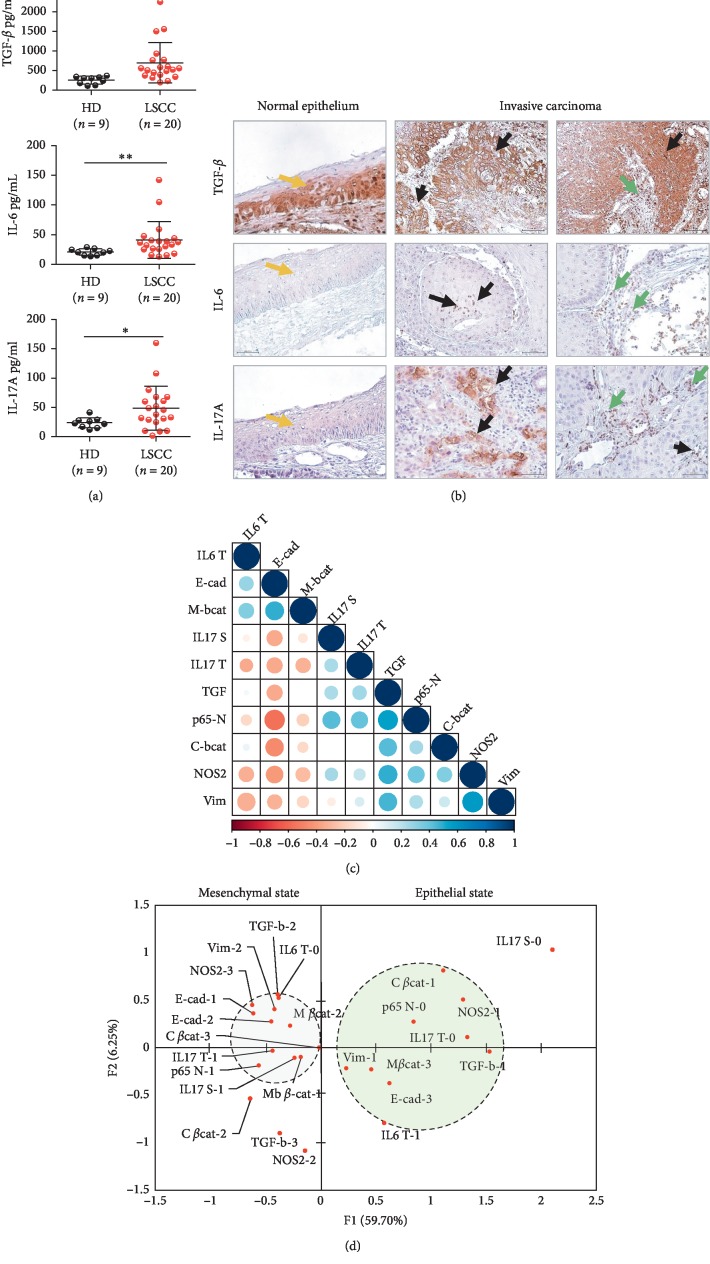
TGF-*β* and IL-17A expression associated with EMT features in invasive LSCC. (a) Plasmatic concentrations of TGF-*β*, IL-6, and IL-17A. ELISA for healthy donors (HD) (*n* = 9) and LSCC patients (*n* = 20) and Mann–Whitney *U* test performed for statistical analysis; data are shown as mean ± SD, ^*∗*^*p* ≤ 0.05; ^*∗∗*^*p* ≤ 0.01; ^*∗∗∗*^*p* ≤ 0.001. (b) Representative TGF-*β*, IL-6, and IL-17A stainings in laryngeal tissues (magnification ×40) (yellow arrow: normal epithelium, black arrow: tumor cells, and green arrow: stomal cells). (c) Correlogram: EMT biomarkers expression levels versus TGF-*β*, IL-6, IL-17A, NOS-2, and NF-*κ*B (p65) scores in invasive LSCC (*n* = 20). (d) MCA of the relation between EMT markers expression, TGF-*β*, IL-6, IL-17A, NOS-2, and NF-*κ*B (p65) scores in invasive LSCC (*n* = 20).

Considering that IL-17A has been shown to mediate EMT in lung cancer [28] and that IL17/Th17 cells would accompany LSCC development [29, 30], we next evaluated the profile expression of IL-17A which may prevail to EMT dependent LSCC invasiveness. Taking into account that TGF-*β* is a determinant inducer of EMT and of IL-17A expression in presence of IL-6 [31], we firstly investigated the pattern of expression of these cytokines in invasive LSCC patients. Our observations showed a significant increase in TGF-*β*, IL-6, and IL-17A levels in patients' plasma (Figure 3(a)). In the tumor biopsies, the expression of these cytokines was notable at the level of the invasion front of the tumors for TGF- *β* and in the tumor (40% cases) and in the stromal areas (100% cases) of the invasive tissues for IL-6 (Figure 3(b)). Interestingly, while IL-17A could be normally detected at the superficial layers of the normal tissues, an enhanced signal accumulated at the level of the tumoral areas of the invasive tissues, in the stroma, and in cells surrounding the blood vessels (Figure 3(b)). TGF-*β* expression in the invasive tumors significantly correlated with a mesenchymal profile (TGF-*β*/Vim: *r* = 0.46, *p*=0.04; Pearson). Interestingly the analysis also showed that IL-17A expression in the tumor and the stromal cells influenced particularly a loss of E-cadherin expression (*r* = −0.35, *p*=0.12; Pearson), thereby pointing to a possible collaborative implication of IL-17 and TGF-*β* to EMT induction (Figure 3(c)).

Considering the finding showing that NF-*κ*B activation is required to IL-17A mediated EMT in lung cancer [28], we next investigated that eventuality in LSCC. We observed that IL-17A signal in the tumor and the stromal areas significantly associated with nuclear NF-*κ*B staining (*r* = 0.46, *p*=0.04; *r* = 0.53, *p*=0.01; Pearson) (Figure 3(c)); therefore, we concluded that IL-17A/NF-*κ*B axis would be involved in LSCC′ epithelial to mesenchymal transition.

To visualize the pattern of expression of the inflammatory mediators influencing EMT, we next performed an MCA. The model recapitulated almost 66% of the total variance and most differences discriminated along the first eingen vector, which described 59.7% of the total inertia. The most discriminating variables were TGF-*β*, IL-17A, and NOS2, which gradually modified NF-*κ*B nuclear status and EMT scores (Figure 3(d)). Collectively, our findings suggested that within LSCC's microenvironment, a strong pattern of association prevailed between the studied soluble mediators of inflammation, NF-*κ*B, and EMT and also suggested that unexplored variables would contribute to that event.

### 3.4. IL-10 and CD68+ Cell Enriched Tumor Microenvironments Associated with the Mesenchymal Shift in Invasive LSCC

**Figure 4:**
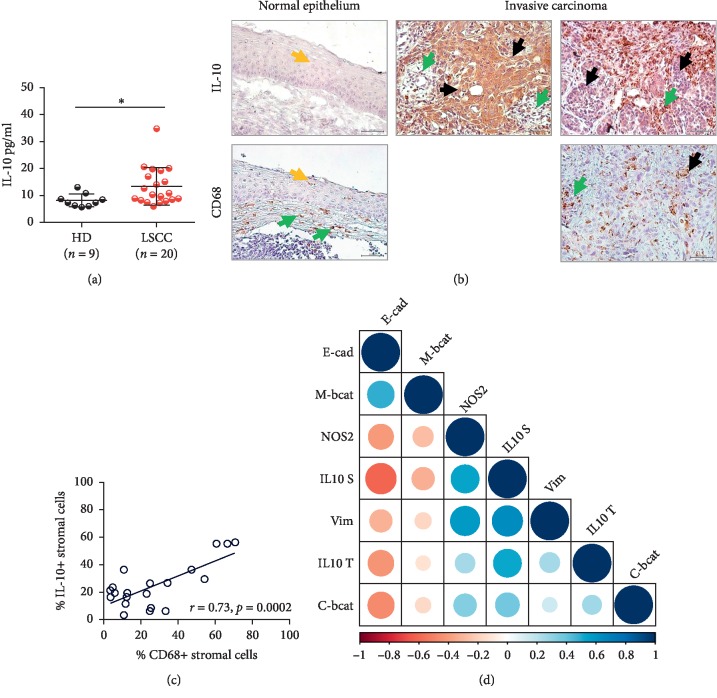
IL-10 expression supports EMT in invasive LSCC. (a) IL-10 plasmatic concentrations by ELISA in healthy donors (HD) (*n* = 9) and LSCC patients (*n* = 20), and Mann–Whitney *U* test performed for statistical analysis; data are shown as mean ± SD, ^*∗*^*p* ≤ 0.05. (b) Representative IL-10 and CD68 stainings in laryngeal tissues (magnification ×40) (yellow arrow: normal epithelium, black arrow: tumor cells, and green arrow: stomal cells). (c) Percentage of stromal cells producing IL-10 was evaluated by IHC and correlated to the percentage of CD68 + stromal macrophage by the Pearson correlation test. (d) Correlogram: EMT biomarkers expression levels versus IL-10 and NOS2 expression in invasive LSCC (*n* = 20).

Considering the reports describing IL-10 upregulation in LSCC and its potential role in EMT induction in cancer [32, 33], we next hypothesized that the cytokine could, in the context of EMT induction in LSCC, be of importance. As described by others, we observed that IL-10 was increased at the systemic level (Figure 4(a)). Importantly, a significant accumulation of the cytokine was noted at the level of the tumor areas and in cells infiltrating the epithelium and the stroma (Figure 4(b)). Further analysis showed that the stromal signal significantly originated from densely infiltrating CD68 + cells (*r* = 0.73, *p*=0.0002; Pearson) (Figure 4(c)). Strikingly, whereas stromal IL-10 expression strongly associated with both vimentin expression and E-cadherin alteration (*r* = 0.64, *p*=0.002; *r* = −0.61, *p*=0.004; Pearson), intratumoral IL-10 solely affected the expression of the epithelial marker (*r* = −0.43, *p*=0.05; Pearson) (Figure 4(d)). Thus, our findings suggest that IL-10 originating from tumor-associated macrophages' (TAMs) and the tumor cells would also likely play a significant role in the acquisition of the invasive features of LSCC.

### 3.5. MMP-9 May Influence EMT in the Context of IL-17A, TGF-*β*, and NF-*κ*B Signaling

**Figure 5:**
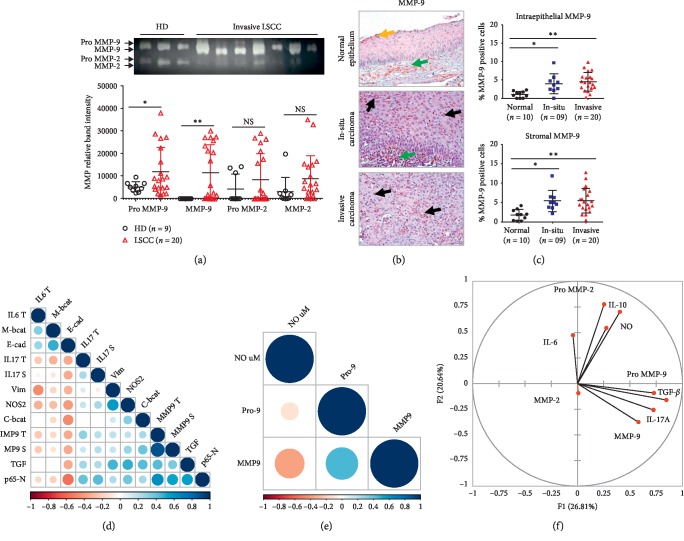
MMP-9 induced EMT occurs in the context of IL-17A, NF-*κ*B, and TGF-*β* signaling. (a) Plasmatic MMP zymography for healthy donors (HD, *n* = 9) and LSCC patients (*n* = 20) and comparison of means of signal intensity activity, depending on the normality test, using a *t*-test or Mann–Whitney test (^*∗*^*p* ≤ 0.05, ^*∗∗*^*p* ≤ 0.01). (b) MMP-9 staining in laryngeal tissues (magnification ×40) (yellow arrow: normal epithelium, black arrow: tumor cells, green arrow: stomal cells). (c) Percentage of MMP-9 positive cells was calculated in epithelial and stromal HPF. One-way ANOVA test followed by Bonferroni's multiple comparison test. (d) Correlogram of EMT markers expression levels, MMP-9, TGF-*β*, IL-17A, NF-*κ*B, and NOS2 in invasive LSCC (*n* = 20). (e) Correlogram: MMP-9 plasmatic levels versus NO_2_^−^ concentrations in invasive LSCC patients (*n* = 20). (f) PCA of plasmatic biomarkers in invasive LSCC (*n* = 20).

Considering that MMP-9 can elicit EMT [17], we next explored that relation in LSCC. MMP activity analysis showed that along with a significant plasmatic expression and activation (Figures 5(a) and 5(b)), a tissular MMP-9 expression (active and inactive MMP) was detectable in the epithelial and the stromal areas of the invasive tissues (Figure 5(c)) (MMP-9 tumor/stroma, *r* = 0.89, *p*=0.001, Pearson). Interestingly, a trend towards a negative link associated E-cadherin with MMP-9 expression in the stroma and the tumor areas (*r* = −0.34, *p*=0.14; Pearson) (Figure 5(d)). To determine if this effect would associate with a pattern of interaction between MMP-9 and the above-explored inducers of EMT, we next performed an unsupervised multivariate analysis (PCA) to take into account the overall structure of the analyzed dataset and the levels of dependence between the analyzed quantitative variables. The analysis recapitulated 47.45% of the total variance and demonstrated that whereas frequent MMP-9 activation associated with IL-17A and TGF-*β* synthesis, increased NOS2-dependent inflammation tended to associate with IL-10 expression (Figure 5(f)). Importantly, Pearson analysis also showed that MMP-9 activation would occur in presence of low plasmatic nitrite levels and concomitantly to NF-*κ*B nuclear translocation (Figures 5(d) and 5(e)).

### 3.6. Pattern Analysis of the EMT Biomarkers Identifies Distinct Immune-Inflammatory Signatures of Tumor Aggressiveness

**Figure 6:**
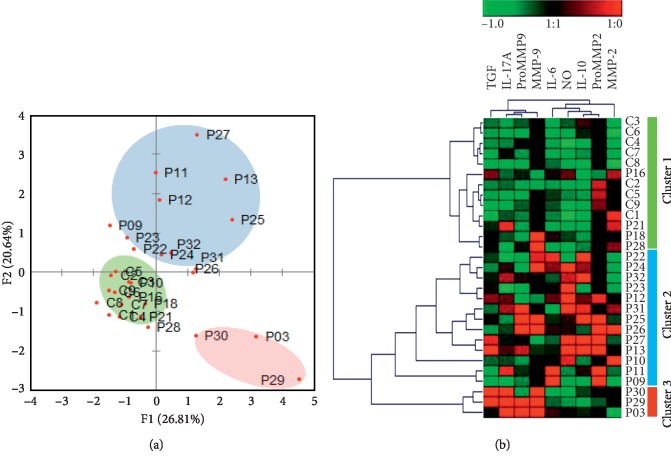
Evaluation of the immune-inflammatory patterns associated with invasive LSCC. (a) Population structure analysis performed by PCA of plasmatic biomarkers in invasive LSCC (*n* = 20). (b) Hierarchical clustering and heat mapping of plasmatic activity patterns in invasive LSCC patients (*n* = 20).

To further characterize the patterns of expression of the soluble inducers of EMT associating with tumor invasiveness, a PCA was performed. 3 distinct clusters of patients separated from the initial population (*n* = 20). Besides, a minor cluster of patients (20%) which shared similarity with the controls, 2 other clusters, representing 65% and 15% of the cohort diverged from the initial population (Figure 6(a)). A heat map analysis of the analyzed biomarkers by unsupervised hierarchical clustering supported the PCA analysis and showed that the distinct clusters of patients segregated based on neighboring biological patterns. The signature found in cluster 1 patients resembled that of healthy individuals; cluster 2 is associated with NO and IL-10-increased expression; and cluster 3 is associated with elevated levels of IL-17A, TGF-*β*, and MMP-9 activity (Figure 6(a)). This suggested that distinct patterns of EMT inducers would associate with LSCC invasiveness.

### 3.7. High IL-17A Expression Associated with a Less Favorable Prognosis in LSCC

**Figure 7:**
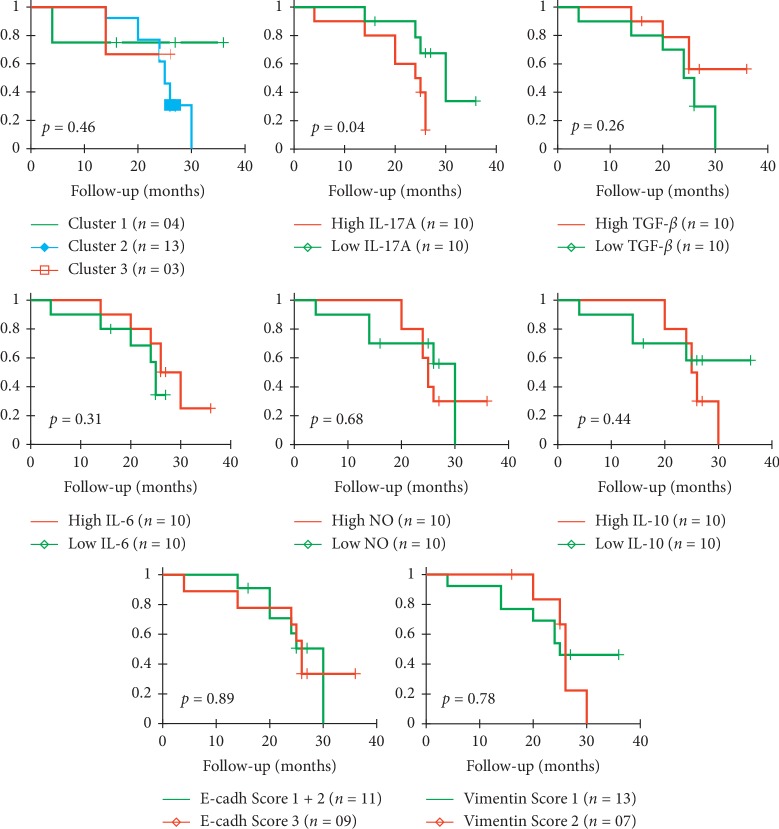
KM survival analysis for LSCC patients (*n* = 20) per clusters and analyzed EMT inducers molecules. Patients' stratification was based on the hierarchical clustering and the median concentrations of the tested biomarkers as cutoffs: IL-17A (42 pg/ml), TGF-*β* (543.5 pg/ml), IL-6 (34.86 pg/ml), IL-10 (10.5 pg/ml), NO (32.69 *μ*M). *p* values were calculated using the log-rank test.

**Figure 8:**
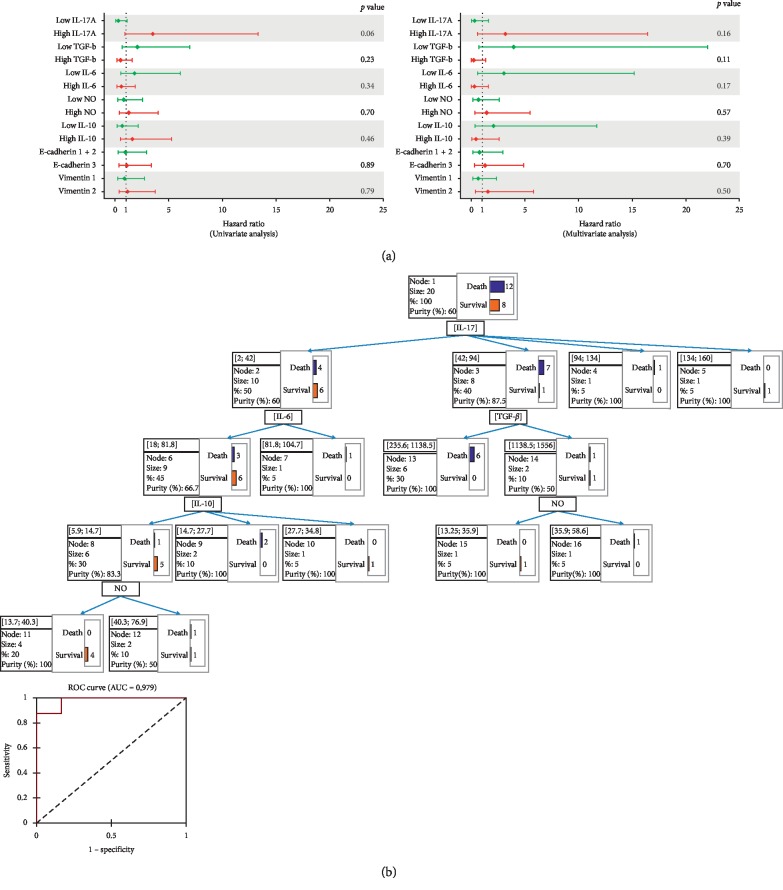
Hazard risk and predictive analysis of conditions of resistance to therapy. (a) Univariate and multivariate analyses of factors associated with risk of death and survival. (b) CHAID regression tree analysis.

To verify if the distinct identified patterns would influence response to therapy, the associated overall survival (OS) and death risk were analyzed. While the OS of the cohort reached 40%, we observed that patients in clusters 2 and 3 tended to associate with shortened survival, as well as those having undifferentiated tumors, high IL-17A (>42pg/ml) or low TGF-*β* (<543.5 pg/ml) plasmatic concentrations (Figure 7). The multivariate Cox regression analysis supported these results and indicated that IL-17A^high^, TGF-*β*^low^, and NO^low^ (<32.69 *μ*M) concentrations would tend to increase patients' death risk. The univariate analysis showed that high IL-17A would constitute a good predictor of poor response to therapy (*p*=0.06) (Figure 8(a)).

Lastly, we performed a Chi-squared automatic interaction decision tree in order to verify if our dataset could predict patients with augmented risk of resistance to therapy. Whereas the specificity of the model reached 91.67% and the sensitivity 87.50%, the area below ROC curve (AUC) indicated an accuracy of 0.979. The predictive model identified IL-17A^high^/TGF-*β*^low^ association as predictors of higher risk of death (6/12, 50%). Besides most of cluster 3, 46.15% of cluster 2 patients associated with that model. The model also identified IL-17A^low^/IL-10^low^ association as the best determinant for response to therapy (5/8, 62.5%). 75% of patients of cluster 1 associated with this model (Figure 8(b)).

## 4. Discussion

In this study, we show, through an ensemble analysis of the soluble mediators of EMT, that heterogeneous immunological patterns drive the development of the invasive features of LSCC. We report that the acquisition of tumor aggressiveness in patients is dependent on close associations prevailing between NOS2 and IL-10 synthesis and between IL-17A, TGF-*β*, and MMP-9 and identifies IL-17A as a possible key predictor of LSCC aggressiveness and resistance to therapy.

At the opposite of Bonavida's report suggesting that transient exposure to NO inhibits NF-*κ*B induced EMT [26], we show the chronic activation of NOS2 in invasive LSCC leads to a positive regulation of both events [27, 34]. This original finding requests further investigations to determine the grounds of these differences and determine possible differences in Snail activation capacities. We speculate that the hypoxic conditions prevalent in LSCC's environment would also be determinant to that process [10, 13].

Our analysis determining that IL-10 associates with EMT induction by NOS2 and NF-*κ*B activity is in line with the reports on the regulatory function of IL-10 on that pathway, which otherwise would lead to NOS2 deleterious function [35, 36]. Our observation showing that exacerbated NOS2 expression associated with tumor necrosis (data not shown) reinforces that interpretation. Considering recent reports, we speculate that the synthesis of NO and IL-10 by tumor-infiltrating macrophages and Tregs [32, 37, 38] would constitute the ground to the inhibition of a protective Th1 response and the condition to the development of IL-10-associated LSCC's loco-regional metastases risk [36, 39]. In turn, in these conditions, coexpression of IL-6 would increase the risk of lymph node metastases by eliciting MDSCs activity and suppressing cytotoxic T-cell function [40, 41].

We also showed that IL-17A synthesis would be strongly involved together with TGF-*β*, and MMP-9 in priming EMT and metastasis. This interpretation is compatible with early reports linking IL-17A in LSCC to tumor angiogenesis and metastasis [22, 29, 30]. Surprisingly, we observed that despite a strong context of Th17 differentiation, IL-17A expression would mostly rely on a tumoral origin; therefore, we hypothesize that distinct conditions prevailing in the milieu, including hypoxia, would be involved in that synthesis [29, 30, 42]. In this context, TGF-*β* would likely contribute to LSCC survival by inhibiting antitumoral T cells [43, 44]. To improve our understanding of the molecular interactions determining the development of either immune-inflammatory patterns supporting EMT, next investigations should analyze the background of NO and IL-10 synthesis in patients, and determine the possible implication of NO-producing Treg cells in the regulation of a Th17/IL-17A response and EMT [45–47].

The univariate analysis of the prognostic value of EMT drivers showed that pretherapy plasmatic IL-17A may constitute a significant determinant of poor prognosis. This observation is in agreement with reports on the function of IL-17A in laryngeal cancer [30] and is concordant with the notion that the cytokine may mediate its pathogenic function, by mediating EMT and metastasis as described for colorectal carcinoma [48]. As IL-17A, as previously reported, did not show to be an independent prognostic factor [30], we report that IL-17A in presence of TGF-*β* would constitute a superior predictive biomarker of LSCC lethality. This result is to our knowledge the first to determine the conditions under which IL-17A would mediate therapeutic failure [30].

## 5. Conclusion

In conclusion, our findings support the notion that LSCC aggressiveness would rely on distinct interactions associating different sets of inflammatory and immunomodulatory molecules supporting EMT and metastasis. Besides requiring to be confronted to a larger cohort, our results underline the necessity to assess the functional relevance of the detected correlations and to explore the cellular and the genetic grounds of the observed tumoral heterogeneity more deeply. Considering the value of IL-17A in predicting therapeutic failure, next investigations should assess in priority if IL-17A functional signaling may also affect disease' development. Taken together, our findings might provide new insight into the influence of immunological regulators of EMT on LSCC development and serve as a ground for the development of new strategies for disease management.

## Data Availability

The raw and derived data used to support the findings of this study are available from the corresponding author upon request.

## Abbreviations

ATRSS: Agency for research development in health
CD: Cluster of differentiation
CHAID: Chi-squared automatic interaction detection
E-cadherin: Epithelial cadherin
ECM: Extracellular matrix
ELISA: Enzyme-linked immunosorbent assay
EMT: Epithelial-mesenchymal transition
HD: Healthy donors
HE: Hematoxylin-eosin
HPF: High-power field
IHC: Immunohistochemistry
IL: Interleukin
IS: Intensity of staining
LSCC: Laryngeal squamous cell carcinoma
MCA: Multiple correspondence analysis
MDSC: Myeloid-derived suppressor cells
MMP: Matrix metalloproteinases
NF-*κ*B: Nuclear factor-kappa B
NO: Nitric oxide
NO2^−^: Nitrites
NOS: Nitric oxide synthase
OS: Overall survival
PCA: Principal component analysis
RNI: Reactive nitrogen intermediates
ROI: Reactive oxygen intermediates
RR: Relative risks
SC: Stained cells
TAMs: Tumor-associated macrophages
Th1: T-helper cell type 1
TGF-*β*: Transforming growth factor beta
UICC: Union for International Cancer Control.
